# Concordance between Clinical and Pathological T and N Stages in Polish Patients with Head and Neck Cancers

**DOI:** 10.3390/diagnostics13132202

**Published:** 2023-06-28

**Authors:** Aldona Chloupek, Joanna Kania, Dariusz Jurkiewicz

**Affiliations:** 1 Department of Cranio-Maxillofacial Surgery, Military Institute of Medicine—National Research Institute, 04-142 Warsaw, Poland; 2 Department of Pathology, Military Institute of Medicine—National Research Institute, 04-142 Warsaw, Poland; jkania@wim.mil.pl; 3 Department of Otolaryngology and Oncology, Military Institute of Medicine—National Research Institute, 04-142 Warsaw, Poland; djurkiewicz@wim.mil.pl

**Keywords:** head and neck cancers, clinical, pathological, T staging, N staging

## Abstract

Background: The TNM (tumor, node, metastasis) staging system is important for the successful treatment of head and neck cancers (HNCs). This study aimed to evaluate the concordance between clinical and pathological T and N stages in patients with HNCs in Poland. Methods: In this single-center retrospective study, clinical and pathological TNM staging data on 203 patients undergoing surgical treatment for HNC between 2011 and 2018 were collected and compared. The study group was classified as underdiagnosed, overdiagnosed, or correctly diagnosed with HNC based on pathological TNM staging. The concordance between clinical and pathological staging was evaluated using the kappa coefficient. Results: Clinical and pathological TNM staging showed concordance in 59.9% of patients for primary tumor (T) and in 79.3% of patients for lymph node (N) classifications. Moderate agreement between the clinical and pathological stages was shown for stage T, while substantial agreement was revealed for stage N. The size and extent of the tumor were underestimated or overestimated in 73 of the 182 patients (40.1%), while lymph node involvement was downstaged in 11 of the 53 patients (20.7%). Conclusions: The disparities between clinical and pathological staging of HNC demonstrate the need for standardization in physical and pathological examinations, as well as radiographic imaging.

## 1. Introduction

Head and neck cancers (HNCs) are defined as a heterogeneous group of tumors that develop in the lip, oral or nasal cavity, pharynx, larynx, paranasal sinuses, and salivary glands [1]. More than 90% of HNCs have squamous histology and arise at the mucosal linings of the upper aerodigestive tract [1,2]. The well-known risk factors for HNC include smoking and excessive alcohol consumption. In addition, the incidence of HNC cases associated with human papillomavirus type 16 has been increasing over the past several decades, especially among younger people [3]. Tumors of the head and neck organs have a generally unfavorable prognosis [4].

It is estimated that HNCs are the seventh most common type of cancer globally, accounting for 6% of all cancers and 5% of cancer-related deaths [2,5]. Every year, around 650,000 to nearly 900,000 new HNC cases are diagnosed worldwide [5,6]. Over the past decades, there has been a steady, almost exponential increase in the incidence of head and neck cancers in Poland. In past years, their percentage among all malignant neoplasms has invariably ranged from 5.5 to 6.2%, which translates to about 5500 to 6000 new cases per year [4]. There is a low level of public awareness of HNCs in Poland. The results of a recent study by Pinkas et al. [5] indicated that more than 70% of respondents declared a lack of knowledge or little knowledge about HNCs. Additionally, Poland is a country with low human papillomavirus vaccination coverage [5,7].

Despite significant clinical advances enabling early diagnosis, HNC treatment remains challenging. About two-thirds of HNC patients are unaware of warning signs, so they are more likely to have advanced disease at the time of diagnosis. This, in turn, increases the risk of postoperative recurrence and metastasis and results in a low 5-year survival rate for HNC of around 50% [8]. Locally advanced disease requires a multidisciplinary approach that includes surgery followed by adjuvant radiotherapy, with or without conventional chemotherapy [9,10]. Recently, immunotherapy with checkpoint inhibitors has been approved for recurrent and metastatic HNC. However, only 15% to 20% of patients may benefit from this therapy [11,12]. Tumor stage, pathological findings, and the specific site of cancer are the leading factors that guide HNC treatment [1].

The stage of the head and neck lesion is critical in the assessment of disease status, prognosis, and therapeutic management. The American Joint Committee on Cancer and Union for International Cancer Control TNM (tumor, node, metastasis) classification is a major tool in current oncology used to describe the patient’s tumor burden [13]. The staging system is based on the characteristics of the tumor at the primary site (T), the degree of regional lymph node involvement (N), and the absence or presence of distant metastases (M) [14]. The final clinical stage (cTNM) is specified based on information from clinical examination, radiologic imaging (ultrasonography, computed tomography, magnetic resonance imaging, and positron emission tomography), biopsy, and other diagnostic tests, while the pathological stage (pTNM) is determined after surgical tumor resection [13,15].

Although the TNM system is widely accepted, it is considered slightly outdated because it does not include data from, for example, immunohistochemical studies or molecular analysis [13]. A lack of predictive power, balance, and differentiation between groups and a failure to account for other tumors, comorbidities, or the patient’s lifestyle were also highlighted [13,16]. The periodic updates to the TNM classification help to incorporate findings from recent studies and nonanatomic prognostic factors to improve outcomes [16,17]. However, it is still difficult to develop a uniform staging system for HNCs because of their histological variation and growth from numerous anatomic sites [17].

Theoretically, the clinical stage (i.e., the preoperative setting determining the selection of therapy) should correlate with pathological staging [17]. However, in patients with HNC, disparities between clinical and pathological TNM stages were reported [18]. One common reason is that imaging methods and other diagnostic tests may not be able to accurately determine the extent of the cancer. For example, small tumors or lymph node involvement may not be visible on images or may be mistaken for other conditions.

Therefore, the aim of this study was to assess the concordance between pathological and clinical T and N staging of HNC in Polish patients.

## 2. Materials and Methods

### 2.1. Study Design

In this single-center retrospective study, we collected the data of 203 patients who had undergone surgery for HNC between 2011 and 2018 in the Department of Cranio-Maxillofacial Surgery of Military Institute of Medicine—National Research Institute in Warsaw, Poland. Patients with primary T1 to T4 oral cancers and who qualified for surgery according to oncology guidelines were included in the study. Patients with recurring cancers who qualified for salvage surgery were excluded from the study. We included all patients for whom data on both clinical and pathological T (*n* = 182) and/or N (*n* = 53) staging were available. Clinical staging was based on palpation and computed tomography. To determine tumor histology after surgery, the tissue was fixed for 24 to 72 h in 10% buffered formalin and embedded in paraffin according to standard protocols. Sections of 2–3 microns were cut from the blocks and stained with Haematoxylin and Eosin. Glass slides were digitalized using Pannoramic 250 FLASH, 3DHISTECH at 20×.

All procedures were conducted in accordance with the Declaration of Helsinki. Ethics approval was not required due to the retrospective study design.

### 2.2. Data Collection

The following data were collected and used for the analysis: age; sex; tumor location; lymph node invasion; tumor histology and grade; surgery type; pathological T, N, and M stages (if the patient had upfront surgery with adjuvant treatment); and protein and albumin levels.

### 2.3. Statistical Analysis

Baseline data were analyzed descriptively. The agreement between clinical and pathological T and N stages was assessed among patients with measurable stages, that is, after excluding patients with Tx and Nx stages, respectively. We calculated the percentages of patients with correct diagnoses (cT = pT, cN = pN), underdiagnoses (cT < pT, cN < pN), and overdiagnoses (cT > pT, cN > pN). The kappa statistic was used to estimate the strength of agreement (kappa of 0–2, slight agreement; 0.21–0.4, fair agreement; 0.41–0.6, moderate agreement, 0.61–0.8; substantial agreement; and 0.81–1, almost perfect agreement) [19]. The agreement plot was used to assess a potential bias for particular T and N stages [20]. All calculations were executed using the R software (v. 4.2.2).

## 3. Results

### 3.1. Characteristics of Patients

**Figure 1:**
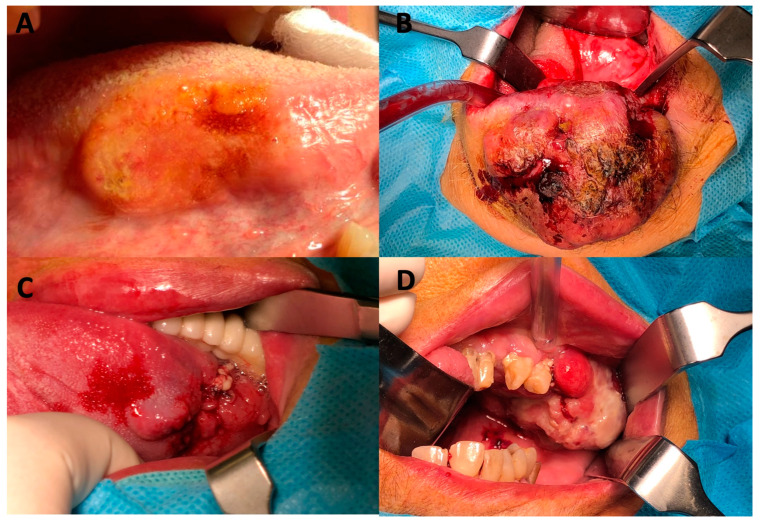
Clinical pictures of selected head and neck cancers. (**A**) tongue cancer; (**B**) lip cancer; (**C**) tongue and floor of mouth cancer; (**D**) gum cancer.

**Figure 2:**
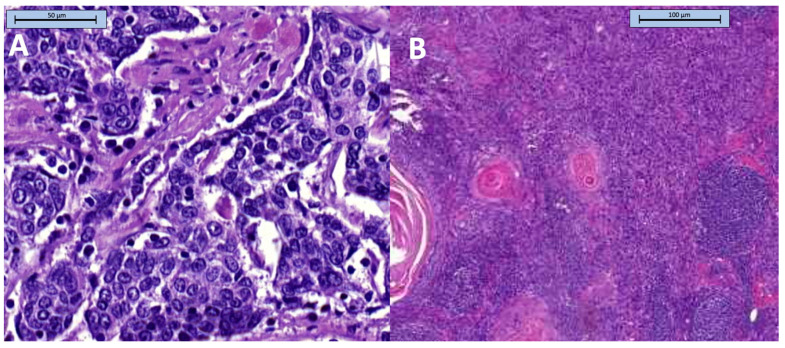
Pathological images of squamous cell carcinoma. (**A**) squamous cell carcinoma metastasis to an intrasalivary lymph node; (**B**) Keratinizing squamous cell carcinoma—metastasis to lymph node. Keratin pearls are present next to the poorly differentiated neoplastic cells and preserved follicular structures.

Data on T and N staging were available for 203 patients. After the exclusion of 21 patients with the Tx stage, there were 182 patients in the T-stage cohort. Similarly, after the exclusion of 150 patients with the Nx stage, there were 53 patients in the N-stage cohort. The median age (64 years) and the proportion of women (~40%) were similar across the cohorts, but the frequency of squamous cell carcinoma (92%) and grade 2 tumors (86%) was higher in the N-stage cohort than in the T-stage cohort (Table 1). The most common types of cancer in both cohorts was tongue cancer and floor of the mouth cancer, but their prevalence was higher in the N-stage cohort (62% vs. 35%). Distant metastases were reported in two patients in the whole study group. The detailed baseline characteristics of the study cohorts are shown in Table 1. Figure 1 and Figure 2 show selected clinical and pathological images of HNCs.

### 3.2. Concordance between Clinical and Pathological T Staging

**Figure 3:**
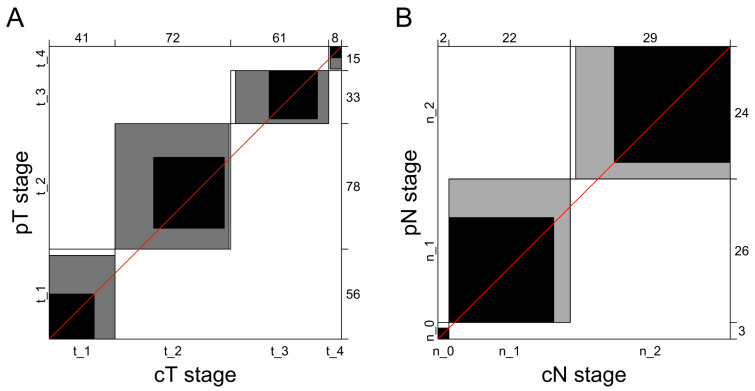
Agreement plots for pathological and clinical T staging (**A**) and N staging (**B**). Numbers denote the amount of patients. Each black rectangle represents the marginal totals of the rows and columns. Shaded boxes represent the agreement based on the diagonal cell frequencies. Diagonal identity line is marked in red.

In the T-stage cohort, the pT1 stage was confirmed in 56 patients (30.8%); pT2, in 78 (42.9%); pT3, in 33 (18.1%); and pT4, in 15 (8.2%). Around 50% of the clinical diagnoses were correct for pT1, pT2, and pT4 stages, and more than 90% of clinical diagnoses were correct for pT3 cancers (Table 2). Overall, clinical and pathological staging showed concordance in 59.9% for T classification (109 of the 182 patients). Tumor extent was underestimated or overestimated in 73 of the 182 patients (40.1%). The agreement between clinical and pathological T staging was moderate (kappa, 0.42; 95% CI, 0.32–0.53). The agreement plot did not show any bias for any of the T stages (Figure 3A).

### 3.3. Agreement between Clinical and Pathological N Staging

In the N-stage cohort, the pN0 stage was confirmed in 3 patients (5.7%); pN1, in 26 (49.0%); and pN2, in 24 (45.3%). Two-thirds (66.7%) of clinical diagnoses were correct for pN0; 73.1%, for pN1; and 87.5%, for pN2 (Table 3). An underestimation or overestimation of the clinical N stage was observed in 11 of the 53 patients (20.7%), yielding an overall diagnostic accuracy of 79.3% in predicting pathological lymph node status. The agreement between clinical and pathological N staging was substantial (kappa, 0.62; 95% CI, 0.42–082). The agreement plot did not show any bias for any of the N stages (Figure 3B).

## 4. Discussion

The clinical assessment of the characteristics of the primary tumor and lymph node involvement in HNC is important for several reasons. First, accurate staging helps physicians to plan appropriate treatment. Advanced tumors require more aggressive treatment, such as a combination of surgery, radiation therapy, and chemotherapy. Conversely, if the cancer is found to be less advanced, less aggressive treatments may be appropriate, potentially minimizing side effects and improving outcomes. Second, accurate staging provides important information about a patient’s prognosis and outcome. Third, accurate staging helps physicians to monitor the recurrence or progression of patients’ cancer(s). Patients with more advanced cancers may require more frequent monitoring and imaging to detect any changes in their disease. Additionally, correct assessment is often an inclusion criterion for enrollment in clinical trials.

The aim of our study was to assess the concordance between pathological and clinical T and N staging of HNC in Polish patients. The last retrospective analysis of the incidence of HNC in Poland shows a slight upward trend in the absolute number of HNCs between 1990 and 2012. Interestingly, a decrease in the incidence of larynx cancers and an increase in the incidence of oropharyngeal were reported [21].

As previously reported, the clinical and pathological T and N stages in HNC may differ, mainly due to inaccuracies in the interpretation of preoperative imaging, the various techniques used for quantitative diagnosis, and the biology of malignant lymph nodes, affecting the uptake of contrast agents [18,22,23,24]. Additionally, histological examinations can be associated with errors, such as inadequate specimen slicing or imprecise lymph node dissection [18,24,25].

In our retrospective analysis, concordance between the pT1, pT2, and pT4 stages and the corresponding cT stages was about 50%. Over 90% of clinical diagnoses were correct for the pT3 stage. Around 66.67% to almost 90% of clinical diagnoses were correct for pN stages, achieving the highest concordance in advanced disease. The clinical examination of a patient can be challenging, as it often involves interpreting and assessing the size of tumors or lymph nodes, but limitations imposed by the patient, such as vomiting reflex or pain, can lead to either over-interpretation or underestimation. It is important to note that clinical TNM (tumor, node, metastasis) staging can only be based on clinical examinations without CT scans. However, to achieve a comprehensive and accurate classification, both clinical examinations and CT scans are necessary. By combining the findings from both approaches, we can make the clinical and pathological TNM classifications as compatible as possible. Therefore, it is incorrect to assume that a complete classification can be achieved based solely on clinical examinations without the aid of CT scans.

In a multicenter prospective study including a large cohort of patients with head and neck squamous cell carcinoma (n = 560), the concordance rate of clinical and pathological staging was 52.2% for the T staging, 53.5% for the N staging, and 54.9% for the overall TNM classification [18]. Another study showed that in 21.8% of the 87 patients with head and neck squamous cell carcinoma, lymph node assessment via computed tomography or magnetic resonance imaging differed from the pathological staging [24], which is in line with our data. A retrospective study by Choi et al. [23] that included patients with oral squamous cell carcinoma revealed that the concordance rate between cT and pT was 87.3%, and between cN and cN, it was 82.5% [23]. Lower rates were obtained by Kreppel et al. [25], who reported good agreement between the clinical and pathological parameters at a level of 62% for T staging and 59% for N staging in a similar group of patients. In 58% of the cases of discordance, the primary tumor was overdiagnosed. In univariate analysis cT, cN, and pT classification had a significant impact on overall survival. In multivariate analysis, only pT and pN-classification had a significant impact on overall survival [25]. In another study that included patients with tongue cancer, the agreement between the clinical and pathological T stage was estimated at 60.6%, while it was 54.5% for the N stage [26].

In our study, T upstaging was frequent in the early stages of HNC (26.9–50% of cases), while an underestimation of tumor size was more common in advanced HNC (53.3%). About one-third of patients with stage pN1 were overdiagnosed on the basis of clinical tumor staging. In one of the three patients with a clinical diagnosis of nodal metastasis (cN2), no tumor was found in dissected lymph nodes (pN0). In contrast to the study by Koch et al. [18], we reported no cases of upstaging among patients with pT3 stage cancer. Clinical T staging is based on tumor size determined by physical and radiographic examination. Imaging examination methods and physical examination have limited accuracy, which may lead to the overestimation and underestimation of the actual stage. According to Choi et al. [23], the main cause of T upstaging is an underestimation of surface tumor dimension (62.5%), followed by deep tumor invasion into the extrinsic muscles of the tongue, which goes undetected by preoperative diagnostic tests (37.5%). On the other hand, errors in N staging are primarily associated with occult single (57.6%) and multiple (42.4%) metastases [23]. Biron et al. [27] emphasized that most T staging discrepancies resulted in upstaging of the disease (from early to advanced stage) in an attempt to ensure that patients were not undertreated [27].

Accurate clinical staging of HNC is challenging due to tumor location, which makes the assessment of tumor size difficult. Comprehensive oropharyngeal examination is hampered by pain and the pharyngeal reflex. Moreover, clinical differentiation of the tumor infiltration depth from the lymphatic tissue of the base of the tongue and the lingual tonsil is complex and requires a histopathological examination. Clinical lymph node assessment during physical examination is also difficult in patients with obesity because the possibility of manual palpation is limited [27]. Clinical staging may be also complicated when the tumor infiltrates the submandibular gland tissue because it is hard to distinguish the enlarged salivary gland from lymph nodes.

Our study has several limitations. First, there is a risk of selection bias, which is inherent to the retrospective study design and depends on the availability of records and the quality of imaging. Additional bias may have occurred due to changes in the accuracy of diagnostic imaging as well as professional experience over the 7-year period of data collection. In addition, numerous observations were excluded due to the lack of T and N staging assessments (21 patients with the Tx stage and 150 patients with the Nx stage), resulting in a small sample size for the study cohorts. Finally, while we assessed discrepancies between clinical and pathological T and N stages, a similar analysis could not have been performed for the M stage due to a small number of patients (only two patients with stage M1 cancer).

## 5. Conclusions

The current study showed a moderate agreement between the clinical and pathological T stages and a substantial agreement between the clinical and pathological N stages in patients with HNC. Our results indicate that upstaging or downstaging of the clinical T stage can be observed in about 40% of patients with HNC, while discrepancies between the clinical and pathological N stages may occur in about 21% of patients. To ensure that T and N staging is adequate, physical, radiological, and pathological examinations should be standardized. Moreover, radiologists and pathologists with expertise in HNC should be involved in the diagnostic process. These are the key factors to ensure correct cancer staging to guide the choice of an appropriate treatment strategy for HNC.

## Figures and Tables

**Table 1:** Patient characteristics.

	Total Cohort	T-Stage Cohort	N-Stage Cohort
Characteristic	*n* = 203	*n* = 182	*n* = 53
Age, years	64 (56, 73)	64 (57, 74)	64 (59, 67)
Women	84 (42)	72 (40)	21 (40)
Histological type			
Squamous cell carcinoma	155 (76)	148 (81)	49 (92)
Other	48 (24)	34 (19)	4 (8)
Grade			
G1	13 (9)	13 (9)	0
G2	112 (78)	110 (78)	38 (86)
G3	19 (13)	18 (13)	6 (14)
Pathological M stage			
Mx	126 (62)	109 (59)	31 (58)
M0	75 (37)	72 (40)	22 (42)
M1	2 (1.0)	1 (1)	1 (2)
Location *			
Tongue	74 (36)	70 (38)	32 (60)
Floor of mouth	65 (32)	63 (35)	33 (62)
Mandible	29 (14)	27 (15)	14 (26)
Lip	29 (14)	28 (15)	4 (8)
Gum	26 (13)	24 (13)	8 (15)
Maxilla	18 (9)	18 (10)	0 (0)
Palette	16 (8)	15 (8)	2 (4)
Cheek	16 (8)	12 (7)	8 (15)
Other	29 (14)	24 (13)	2 (4)
Surgery type			
Resection	196 (97)	176 (97)	52 (100)
Biopsy	6 (3)	5 (3)	1 (2)
Nodes dissection			
SND	84 (41)	78 (43)	33 (62)
RND	55 (27)	53 (29)	31 (58)
MRND	35 (17)	35 (19)	20 (38)
ERND	4 (2)	3 (2)	2 (4)
Adjuvant treatment			
None	137 (68)	121 (66)	24 (45)
Radiation	57 (28)	54 (30)	25 (47)
Radiation and chemotherapy	7 (3)	6 (3)	3 (6)
Chemotherapy	2 (1)	1 (1)	1 (2)
Plastic surgery			
Local	121 (60)	106 (58)	30 (57)
Other	58 (28)	56 (31)	19 (36)
None	24 (12)	20 (11)	4 (7)

Data are presented as median (interquartile range) or number (%) of patients. Percentages may not total 100 due to rounding. * In one patient, the tumor could have more than one location. ERND—extended radical neck dissection; MRND—modified radical neck dissection; RND—radical neck dissection; SND—selective neck dissection.

**Table 2:** Pathological and clinical T stages.

Pathological Stage	Total, *n* (%)	cT1, n	cT2, n	cT3, n	cT4, n	Correct Diagnosis, *n* (%)	Underdiagnosis, *n* (%)	Overdiagnosis, *n* (%)
pT1	56 (30.8)	28	24	3	1	28 (50)	0 (0)	28 (50)
pT2	78 (42.9)	13	44	21	0	44 (56.4)	13 (16.7)	21 (26.9)
pT3	33 (18.1)	0	3	30	0	30 (90.9)	3 (9.1)	0 (0)
pT4	15 (8.2)	0	1	7	7	7 (46.7)	8 (53.3)	0 (0)

**Table 3:** Pathological and clinical N stages.

Pathological Stage	Total, *n* (%)	cN0, n	cN1, n	cN2, n	Correct Diagnosis, *n* (%)	Underdiagnosis, *n* (%)	Overdiagnosis, *n* (%)
pN0	3 (5.7)	2	0	1	2 (66.7)	0 (0)	1 (33.3)
pN1	26 (49.0)	0	19	7	19 (73.1)	0 (0)	7 (26.9)
pN2	24 (45.3)	0	3	21	21 (87.5)	3 (12.5)	0 (0)

## Data Availability

The data that support the findings of this study are available from the corresponding author upon reasonable request.
